# Prevalence of Malocclusion in 3- to 5-Year-Old Children in Shanghai, China

**DOI:** 10.3390/ijerph14030328

**Published:** 2017-03-22

**Authors:** Xinhua Zhou, Ying Zhang, Yan Wang, Hao Zhang, Li Chen, Yuehua Liu

**Affiliations:** 1 Department of Orthodontics, School & Hospital of Stomatology, Tongji University, Shanghai Engineering Research Center of Tooth Restoration and Regeneration, Shanghai 200072, China; joyce2771536@hotmail.com; 2 Department of Preventive Dentistry, Shanghai Stomatological Hospital, Shanghai 200001, China; zhangyingcmu@vip163.com (Y.Z.); serene2000@163.com (Y.W.); haozhang18@foxmail.com (H.Z.); 3 Department of Orthodontics, Shanghai Stomatological Hospital, Shanghai 200001, China; chli09132000@163.com

**Keywords:** primary dentition, malocclusion, epidemiology, prevalence

## Abstract

The aim of the present study was to obtain the prevalence of malocclusions in preschool children in Shanghai, China. A cross-sectional survey was conducted among 2335 children aged 3–5 years from kindergartens. Several occlusal parameters were clinically assessed, including second deciduous molar terminal plane, canine relationship, degree of overjet and overbite, anterior and posterior crossbite, and the presence or absence of physiologic spaces and crowding. All parents of subjects were asked to fill in the oral health knowledge questionnaires. The prevalence of malocclusion in primary dentition in Shanghai was 83.9%, and no significant differences were found in genders. Data showed that the prevalence of deep overbite (63.7%) was the highest in children with malocclusion, followed by deep overjet (33.9%), midline deviation (26.6%), anterior crossbite (8.0%) and anterior crowding (6.5%). The results revealed a high prevalence of malocclusion in primary dentition in children aged 3–5 years old of Shanghai, especially in vertical anomalies. The need for preventive orthodontic therapy is extremely desired and oral health education about malocclusion should be strengthened.

## 1. Introduction

As an improving quality of living, people are hoping to own an aesthetically pleasing appearance. This evolution has driven many industries to satisfy their clients’ aesthetic needs, including dentistry. The rapid development of China’s economy has resulted changes in diet, making people consume more refined food. However, this dietary change results in insufficient jaw growth [1]. Malocclusion is a disorder of the craniofacial complex that affects the development of dental maxillofacial region and masticatory function [2]. Serious malocclusion may cause both psychological and physiological conditions. Therefore, it is important to find out the incidence of various malocclusions and corresponding methods to prevent or correct them.

Early intervention for children in, or before, the peak of growth and development can reduce not only the prevalence of malocclusion or the severity in permanent dentition, but also the psychological impact. A number of studies had investigated the prevalence of malocclusions in the primary dentition in different countries and populations, with prevalence values ranging from 21.0% to 88.1% [3,4,5,6,7,8,9,10]. A study about Chinese people from 1956 to 1960 showed the prevalence ranging from 29.33% to 48.87%. A national survey conducted by Chinese Stomatological Association (CSA) in 2000 concluded the prevalence of malocclusion as 51.84% in Chinese children [11]. Some studies suggested that malocclusions were also related with bad oral habits, such as mouth-breathing and non-nutritive sucking habits [1,12].

The aim of the present study was to evaluate the prevalence of malocclusion in the primary dentition and bad oral habits of preschoolers in the city of Shanghai, in order to provide an epidemiological reference for the development of early intervention and prevention of the occurrence of malocclusion. 

## 2. Materials and Methods

### 2.1. Study Design and Study Population

**Figure 1:**
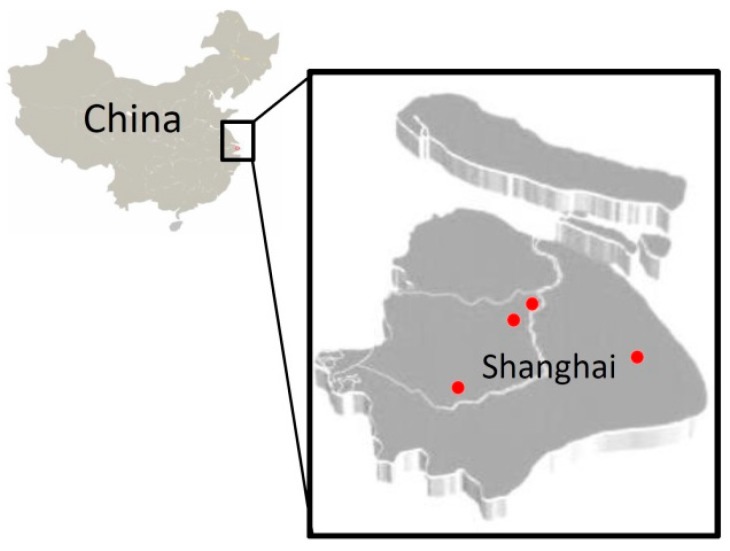
The locations where the study took place.

A multistage, stratified sampling method was applied to obtain a representative sample of preschoolers, and we selected four districts (Hongkou District, Putuo District, Pudong District, and Minhang District) by probability proportional to size sampling (PPS) (Figure 1). The sample was composed of 2335 children (1247 boys and 1088 girls) aged 3 to 5 years from 12 kindergartens. The included children were studied in the kindergartens which were sampling surveyed and we also obtained their parents’ or guardians’ informed consent before examination was initiated. The exclusion criteria were the presence of permanent teeth, loss of any primary teeth, dental caries that affected the judgment, orthodontic treatment history, tooth agenesis, and other congenital malformation (such as cleft lip/palate) or severe illness and children unable to cooperation. The survey was conducted from January to June, 2016. This study was approved by the Ethics Committee of Shanghai Stomatological Hospital (2015-0012).

### 2.2. Questionnaire

The investigation was composed by an anamnestic questionnaire and oral examination measurements without radiograms, which were mostly based on the WHO basic methods for conducing oral health surveys [13].

The questionnaires were completed by parents under the dentists’ instruction. The first section was about general information, such as age and gender of the child. The second section contained ten questions about the children’s oral habits and parents’ awareness of oral health.

### 2.3. Dental Examination

The oral examination was carried out by five calibrated trained orthodontic dentists. A pilot study on 50 children was conducted before beginning the present investigation to ensure the accuracy of diagnosis and to standardize the procedures, and substantial inter-examiner reliability was found (Kappa agreement value >0.9). The children were examined in schools’ infirmaries. Each child was checked with a pair of disposable latex gloves and a disposable mouth mirror.

Following items were included in the oral examination:

#### 2.3.1. Sagittal Anomalies

- Deciduous canine relationship: Equal to Angel’s classification. The canine relationship was recorded as class II or class III, if it was class I on one side and class II or class III on the other. Children with class II canine relation on one side and class III on the other side were recorded as mixed.
- Terminal plane relationship of the second primary molars: The relationship of the distal surface between the upper and lower second deciduous molar including three types (flush type, mesial type and distal type). The relationship of molars and canines were recorded on the basis of bilateral occlusion.
- Maxillary overjet: This was measured from the palatal surface of the mesial corner of the most protruded maxillary incisor to the labial surface of the corresponding mandibular incisor. (0 mm: edge-to-edge; >3 mm, ≤5 mm: mild; >5 mm, ≤8 mm: moderate; >8 mm: severe).
- Mandibular overjet (anterior crossbite): This was recorded when one or more of the maxillary incisors or canine occluded lingual to the mandibular incisors.

#### 2.3.2. Vertical Anomalies

- Overbite: This was graded according to coverage of the mandibular incisor by the most protruded fully erupted maxillary incisor. (<1/2: normal; >1/2, ≤3/4: mild; >3/4, <1: moderate; all cover: severe).
- Open bite, anterior (<3 mm: mild; >3 mm, ≤5 mm: moderate; >5 mm: severe).

#### 2.3.3. Transversal Anomalies

- Posterior crossbite: This was recorded when one or more of the maxillary primary molars occluded the lingual to the buccal cusps of the opposing mandibular teeth.
- Scissors bite: This was recorded when one or more maxillary primary molars occluded the buccal to the buccal surfaces or the lingual to the lingual surfaces of the corresponding mandibular teeth.
- Midline displacement.

#### 2.3.4. Space Discrepancies

- Crowding (anterior, posterior): >0, ≤2 mm: mild; >2 mm, ≤4 mm: moderate; >4 mm: severe
- Spacing: >0, ≤2 mm: mild; >2 mm, ≤4 mm: moderate; >4 mm: severe

#### 2.3.5. Others

- Dental arch shape: triangular; U-shape; square-shape
- Tonsil: normal; antiadoncus I^°^; antiadoncus II^°^; antiadoncus III^°^
- Temporomandibular joint disorder
- Nasal ventilation
- Mandibular plane angle

Anterior crossbite, posterior crossbite, deep overbite (>1/2), deep overjet (>3 mm), anterior open bite, anterior edge-to-edge, posterior scissor bite, and crowding (>2 mm) all indicated malocclusion. The preschool children who exhibited at least one of these conditions were classified with malocclusion.

### 2.4. Statistical Analysis

Data were recorded in a spreadsheet computer program (Microsoft Excel 2010, Microsoft Corp., Redmond, WA, USA). SPSS 22.0 software (SPSS, Chicago, IL, USA) was used for analyses. The results of intra‑examiner reliability were tested using the kappa agreement statistic method.

The prevalence of malocclusion was reported by age and gender, and in total. The chi-square test was applied to determine the statistical associations between the independent variables and the malocclusion variable. For all tests, significant difference was assumed when the *p* value is < 0.05. The clinical registrations were based on the method evolved by the Angle’s classification, which has been used in many studies [14].

## 3. Results

The present study showed that 16.1% of children had dentitions without any irregularity and 83.9% had different degrees of anomaly (Table 1). There was no significant difference found in genders. Data showed that prevalence of deep overbite (63.7%) was the highest in children with malocclusion, followed by deep overjet (33.9%), midline deviation (26.6%), anterior crossbite (8.0%), and anterior crowding (6.5%) (Table 2, Table 3, Table 4 and Table 5).

The study revealed that the most common molar relationship at the 3–5 years of age was the flush terminal plane (38.7%), followed by mesial step (38.5%) (Table 2). With respect to the canine relationship, the normal type was observed as 57.0%, and the distal type as 32.4% (Table 2).

**Figure 2:**
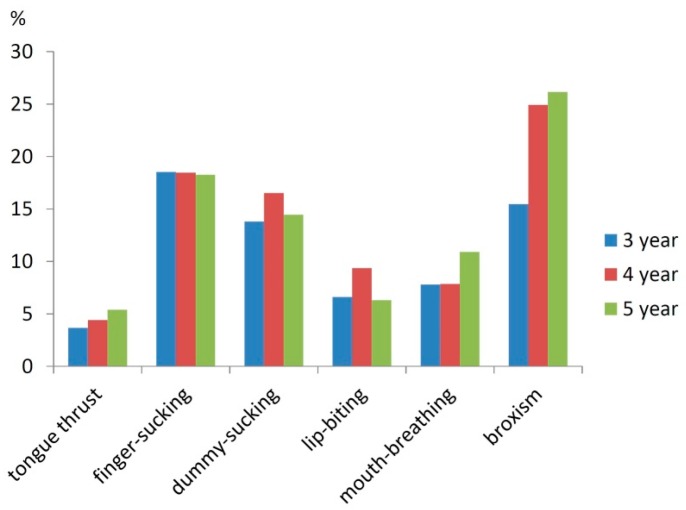
Composition ratio of inadequate oral habits at each age.

There were 63.4% children with bad oral habits, 32.7% of them had sucking habits, and 48.5% parents had no awareness about orthodontic treatments (Figure 2).

## 4. Discussion

The results showed that prevalence of malocclusion from 3 to 5 years old was 83.9%, which is much higher than 51.84% for children with primary dentition in China [11]. The prevalence was also different from that reported in studies which were carried out in different countries, such as 26.0% reported in India and 42.0% to 74.7% in Germany [6,7,15]. These differences may be due to the different methodology used by the authors, or different subjects and decades. Race, living environment, and eating habits were different in the various regions, which may affect the incidence of malocclusion.

Our study on the Shanghai population showed that distribution of flush terminal molar relation was 38.7% on both side. A study by Infante pointed out that the distal step molar relationship decreased with the increase of age [16]. Other studies by Nanda et al. and Ravn indicated that the distal step molar relationship was invariably maintained throughout the primary dentition stage and always transferred unchanged to the permanent dentition [3,17]. The research done by Ravn was a longitudinal study, to ensure the result was more reliable. With regard to the flush and mesial terminal plane, Onyeasoet et al. found out most of them developed into Angle class I in the permanent dentition [18]. The present study was a cross-sectional study which inevitably imposed limitations on the estimation. Further longitudinal studies are needed to obtain the changes in occlusal pattern from the deciduous dentition to permanent dentition.

The prevalence of the class I canine relationship in our study was 57.0%. Children with a class II canine relationship reached 32.4%, which was much lower than 45% in British children [19], but was similar to 31.6% in the Danish children [17]. The difference could be caused by small sample size in the former study, which may enlarge the sampling error to misunderstand the actual situation.

Table 2 suggested that the two more prevalent types of anomalies were dental space and deep overbite, which is consistent with previous studies [14,20]. Primate space and leeway space are normal in deciduous dentition. A study published by Center of Human Development at the University of Michigan showed the sum of mesiodistal diameters of primary teeth was 6, shorter than that of permanent teeth in the maxillary [21]. The permanent dentition may be crowded if there are no spaces in the deciduous dentition. However, this theory is questionable since Baume showed that nine of 16 individuals with no interdental spaces in the primary dentition did not exhibit crowding in the permanent dentition [22]. This indicates that leeway space does not necessarily solve the problem of crowding. More longitudinal studies should be conducted to determine this in the future. Hence, dental space was temporarily not classified as malocclusion in our study. The prevalence of crowding (6.5%) was much lower than in Colombia (52.1%) [14]. This may be due to the cutoff value of the latter article being more than 0 mm.

As interdental space was removed from malocclusion in primary dentition, the most prevalent type of malocclusion became deep overbite (63.7%), followed by deep overjet (33.9%). These results were similar to those reported in previous studies [7,23,24,25]. The prevalence of deep overbite was high in deciduous dentition and increased to the late mixed dentition, which may be explained by the common use of extraction of deciduous molars, a procedure that will usually result in collapsed dentition. Full eruption of the premolars and second molars could stabilize the occlusion, and the prevalence of deep overbite may decrease in the permanent dentition. During craniofacial growth, the mandible will rotate in a backward direction [26], while the overjet will decrease.

In the present study, 33.9% of the children showed deep overjet, which was higher than 29.7% in Brazil [27] and 26.0% in Finnish subjects [23]. These differences may be caused due to the use of different methodologies by the authors, who considered an accentuated overjet to be greater than 3 mm, in comparison to the 2 mm used in the present study for the determination of this condition.

The prevalence of anterior crossbite was 8.0% in the present study, which was more than the Saudi (1.7%) and the British (1.0%). However, it was similar to the prevalence in the Finnish (8%) [28] and African-Americans (5%) [29]. Previous studies on Americans and Europeans indicated the incidence of posterior crossbite in the primary dentition ranging from 7.2% to 20.81% [5,7]. Another study showed that it was one of the most prevalent malocclusions in the primary and early mixed dentitions [30]. However, in the present study, the prevalence of posterior crossbite was much lower, which meant only 6 in 2334 children had posterior crossbite. Our result was similar to the result found in India (0.4%) [31]. It observed that Caucasians generally showed higher incidence rate of posterior crossbite than Africans and Asians [17,32,33]. The different prevalence of posterior crossbite between different regions may be caused by the difference in prevalence of sucking habits. Three studies on posterior crossbite associated this alteration to finger-/dummy-sucking habits which, in the present study, was 32.7% [5,25,34]. The children who adopted such habits tend to have a greater chance of exhibiting posterior crossbite than those that did not. However, research shows the scientific evidence could not confirm what type of malocclusion is associated with non-nutritive sucking habits [35].

Malocclusion not only destroys aesthetics, but also creates functional problems. Studies suggest that deep overjet and anterior open bite were predisposing factors of dental trauma [36,37,38]. Young children start to crawl, walk, run, and fall, and take up high-risk activities when they grow up, such that dental injuries and dislocated teeth are common [39]. Cross-bite is unlikely to lead to the development of oral disease, but dysfunction can arise from the resulting impairment of mastication [40]. 

The definition of early treatment it a treatment which is started in the primary or mixed dentition to enhance the dental and skeletal development before the eruption of permanent dentition [41]. These early therapeutic methods are usually brief and simple, which elicits little cooperation from patients and their parents. Early treatments could prevent the malocclusion from worsening and greatly simplify subsequent orthodontic treatment. Malocclusion caused by bad oral habits, such as open bite caused by tongue protrusion, can be corrected by tongue crib appliance [42]. It seems that some early interventions are needed in order to prevent the malocclusion from worsening and obtain a well-balanced dental and skeletal development.

The present study indicated a high prevalence of malocclusion for 3 to 5-year-old children in Shanghai, which should be taken seriously. However, it is also important to construct a more precise definition of primary dentition malocclusion such that common standards are defined for further studies. The change of malocclusion with increasing age could not be indicated due to our cross-sectional research, and a further longitudinal study is needed to determine this.

## 5. Conclusions

The present study offers evidence that malocclusion is a remarkable problem in Shanghai’s preschool children. The prevalence of malocclusion was high among the children analyzed and increased significantly in recent 17 years, suggesting that malocclusion is a public health problem worthy of note in the Chinese population. 

## Figures and Tables

**Table 1:** Descriptive analyses of demographic characteristics of sample.

Age & Gender	*n*	Normal Occlusion		Malocclusion		*p*
		*n*	%	*n*	%	
Age						0.121
3	846	124	14.7	722	85.3	
4	728	112	15.4	616	84.6	
5	761	139	18.3	622	81.7	
Gender						0.886
Boys	1247	199	53.1	1048	53.5	
Girls	1088	176	46.9	912	46.5	
Total	2335	375	16.1	1960	83.9	

Chi-square test: *p* > 0.05.

**Table 2:** The composition and prevalence of sagittal occlusal characteristic.

Sagittal Occlusal Characteristic	Age 3 (Year)		Age 4 (Year)		Age 5 (Year)		Total		
	*n*	%	*n*	%	*n*	%	*n*	%	
Canine relationship									
Normal (class I)	496	58.6	415	57.0	419	55.1	1330	57.0	
Distal (class II)	254	30.0	239	32.8	264	34.7	757	32.4	
Mesial (class III)	83	9.8	69	9.5	75	9.9	227	9.7	
Mix ^1^	13	1.5	5	0.7	3	0.4	21	0.9	
Second deciduous molar terminal plane									
Bilateral symmetry	751	88.8	643	88.3	671	88.2	2065	88.4	
Flush	332	39.2	265	36.4	306	40.2	903	38.7	
Distal	70	8.3	129	17.7	65	8.5	264	11.3	
Mesial	349	41.3	249	32.7	300	39.4	898	38.5	
Deep overjet	294	34.8	264	36.3	233	30.6	791	33.9	
Edge to edge	16	1.9	15	2.1	23	3.0	54	2.3	
Mild (>3 mm, ≤5 mm)	222	26.2	202	27.7	183	24.0	607	26.0	
Moderate (>5 mm, ≤8 mm)	61	7.2	58	8.0	43	5.7	162	6.9	
Severe (>8 mm)	11	1.3	4	0.5	7	0.9	22	0.9	
Anterior crossbite	68	8.0	49	6.7	70	9.2	187		8.0

Mix ^1^: Child with class II canine relation on one side and class III on the other side was recorded as mixed.

**Table 3:** The composition and prevalence of vertical anomalies.

Vertical Anomalies	Age 3 (Year)		Age 4 (Year)		Age 5 (Year)		Total	
	*n*	%	*n*	%	*n*	%	*n*	%
Deep overbite	532	62.9	499	68.5	457	60.1	1488	63.7
Mild (>1/2, ≤3/4)	204	24.1	172	23.6	144	18.9	520	22.3
Moderate (>3/4, <1)	202	23.9	224	30.8	185	24.3	611	26.2
Severe (all cover)	126	14.9	103	14.1	128	16.8	357	15.3
Open bite	5	0.6	3	0.4	2	0.3	10	0.4
Moderate (>3 mm, ≤5 mm)	4	0.5	3	0.4	1	0.1	8	0.3
Severe (>5 mm)	1	0.1	0	0.0	1	0.1	2	0.1

**Table 4:** The composition and prevalence of transversal anomalies.

Transversal Anomalies	Age 3 (Year)		Age 4 (Year)		Age 5 (Year)		Total	
	*n*	%	*n*	%	*n*	%	*n*	%
Midline displacement	224	26.5	190	26.1	206	27.1	620	26.6
Posterior Teeth Malocclusion								
Posterior crossbite	1	0.1	5	0.7	0	0.0	6	0.3
Edge to edge	0	0.0	0	0.0	0	0.0	0	0.0
Scissors bite	3	0.4	2	0.3	2	0.3	7	0.3
Opposite Scissors bite	0	0.0	1	0.1	0	0.0	1	0.0

**Table 5:** The composition and prevalence of space discrepancies.

Anterior Teeth Malocclusion	Age 3 (Year)		Age 4 (Year)		Age 5 (Year)		Total	
	*n*	%	*n*	%	*n*	%	*n*	%
Crowding	46	5.4	33	4.5	72	9.5	151	6.5
Maxillary	38	4.5	17	2.3	23	3.0	78	3.3
>2 mm, ≤4 mm	36	4.3	15	2.1	19	2.5	70	3.0
>4 mm	2	0.2	2	0.3	4	0.5	8	0.3
Mandibular	59	7.0	26	3.6	61	8.0	146	6.3
>2 mm, ≤4 mm	56	6.6	21	2.9	55	7.2	132	5.7
>4 mm	3	0.4	5	0.7	6	0.8	14	0.6
Spacing	349	41.3	348	47.8	349	45.9	1046	44.8
Maxillary	306	36.2	317	43.5	297	39.0	920	39.4
>2 mm, ≤4 mm	168	19.9	173	23.8	157	20.6	498	21.3
>4 mm	138	16.3	144	19.8	140	18.4	422	18.1
Mandibular	211	24.9	204	28.0	221	29.0	636	27.2
>2 mm, ≤4 mm	144	17.0	133	18.3	152	20.0	429	18.4
>4 mm	67	7.9	71	9.8	69	9.1	207	8.9
